# Dendritic Cell Targeting Using a DNA Vaccine Induces Specific Antibodies and CD4^+^ T Cells to the Dengue Virus Envelope Protein Domain III

**DOI:** 10.3389/fimmu.2019.00059

**Published:** 2019-01-29

**Authors:** Arthur Baruel Zaneti, Marcio Massao Yamamoto, Fernando Bandeira Sulczewski, Bianca da Silva Almeida, Higo Fernando Santos Souza, Natália Soares Ferreira, Denicar Lina Nascimento Fabris Maeda, Natiely Silva Sales, Daniela Santoro Rosa, Luís Carlos de Souza Ferreira, Silvia Beatriz Boscardin

**Affiliations:** ^1^Department of Parasitology, Institute of Biomedical Sciences, University of São Paulo, São Paulo, Brazil; ^2^Department of Microbiology, Institute of Biomedical Sciences, University of São Paulo, São Paulo, Brazil; ^3^Department of Microbiology, Immunology and Parasitology, Federal University of São Paulo (UNIFESP/EPM), São Paulo, Brazil; ^4^Institute for Investigation in Immunology (iii)-INCTiii, São Paulo, Brazil

**Keywords:** dengue fever, dendritic cells, envelope protein domain III, single-chain Fv antibody, DNA vaccine

## Abstract

Dengue fever has become a global threat, causing millions of infections every year. An effective vaccine against all four serotypes of dengue virus (DENV) has not been developed yet. Among the different vaccination strategies available today, DNA vaccines are safe and practical, but currently induce relatively weak immune responses in humans. In order to improve immunogenicity, antigens may be targeted to dendritic cells (DCs), the main antigen presenting cells and orchestrators of the adaptive immune response, inducing T and B cell activation. It was previously shown that a DNA vaccine encoding a fusion protein comprised of an antigen and a single-chain Fv antibody (scFv) specific for the DC endocytic receptor DEC205 induced strong immune responses to the targeted antigen. In this work, we evaluate this strategy to improve the immunogenicity of dengue virus (DENV) proteins. Plasmids encoding the scFv αDEC205, or an isotype control (scFv ISO), fused to the DENV2 envelope protein domain III (EDIII) were generated, and EDIII specific immune responses were evaluated in immunized mice. BALB/c mice were intramuscularly (i.m.) immunized three times with plasmid DNAs encoding either scDEC-EDIII or scISO-EDIII followed by electroporation. Analyses of the antibody responses indicated that EDIII fusion with scFv targeting the DEC205 receptor significantly enhanced serum anti-EDIII IgG titers that inhibited DENV2 infection. Similarly, mice immunized with the scDEC-EDIII plasmid developed a robust CD4^+^ T cell response to the targeted antigen, allowing the identification of two linear epitopes recognized by the BALB/c haplotype. Taken together, these results indicate that targeting DENV2 EDIII protein to DCs using a DNA vaccine encoding the scFv αDEC205 improves both antibody and CD4^+^ T cell responses. This strategy opens perspectives for the use of DNA vaccines that encode antigens targeted to DCs as a strategy to increase immunogenicity.

## Introduction

Dengue virus (DENV) is the causative agent of dengue fever, an infection that has become a serious public health issue. In the last decades, the alarming increase in the number of cases [50–100 million per year, (1)] and also the increase in the incidence of more severe clinical forms of the disease (like dengue hemorrhagic fever, DHF or dengue shock syndrome, DSS) led the World Health Organization to prioritize the development of a vaccine against dengue (2). DENV is transmitted to humans by the bite of mosquitoes of the genus *Aedes* (such as *Aedes aegypti* and *Aedes albopictus*) infected with one of the four virus serotypes (DENV 1–4) (3).

The virus genome is translated into a polyprotein which is processed by virus and host proteases to produce three proteins that make up the viral particle: capsid (C), pre-membrane/membrane (prM/M) and envelope (E), and seven other non-structural proteins, NS1, NS2a, NS2b, NS3a, NS4a, NS4b, and NS5 (3). The E protein plays an important role in the protective immunity against DENV as it contains the majority of epitopes that elicit neutralizing antibodies (4–6). This protein can be divided into three domains: the central domain (EDI), the domain responsible for dimerization containing the fusion peptide (EDII), and the domain that binds to the surface cell receptor (EDIII) (2). EDIII has been extensively used in vaccine development for its ability to induce antibodies able to block DENV infection (7–9). In addition to neutralizing antibodies, T cell responses also play a relevant role in the development of protection. T cells limit the spread of viral infection because they kill infected cells and secrete pro-inflammatory cytokines (10, 11). IFNγ-secreting Th1 and CX3CR1^+^ cytotoxic CD4^+^ T cells are also associated with protection (12, 13).

Dendritic cells (DCs) are central for immunity induction, activating both T and B cells. These cells are excellent antigen presenting cells (APCs) because of their ability to acquire different antigens (either by pinocytosis, endocytosis, or phagocytosis), when compared to other cell types such as macrophages and B cells (14). To accomplish their role as APCs, DCs express a large number of extra and intercellular receptors that are responsible for their ability to “sense” the environment. When they encounter an antigen in the context of infection/inflammation, DCs undergo a maturation process that results in the up-regulation of co-stimulatory and MHCII molecules, and increases their ability to present antigens in the context of MHC I and II (15).

The last decades have proven to be extremely prolific for the study of DC biology and function, as different DC subsets were identified both in humans and in mice (16). Each subset is normally characterized by the expression of different surface markers. The DEC205 is an endocytic C-type lectin receptor expressed by murine and human DCs in different organs (17–20) that uptakes antigens and directs them to MHCII rich late endosomes, increasing antigen presentation to CD4^+^ T cells (21). In mice, DEC205^+^ DCs are resident in the T cell zone of lymphoid organs, and also express the CD8α marker (22). The DEC205^+^CD8α^+^ DCs were involved in the uptake of dying cells, and in the resistance to some viral infections (23–25). Antigens derived from different pathogens have been targeted to DEC205^+^CD8α^+^ DCs using chimeric anti-DEC205 monoclonal antibodies (mAbs) genetically fused to them, administered in the presence of a DC maturation stimulus (26–34).

The use of chimeric mAbs to target antigens to different endocytic receptors has become more spread, and this concept was employed in the development of more effective DNA vaccines. These vaccines are usually safe, cheap, easy to produce but may fail to induce strong immune responses, especially in humans (35). An improvement in the immune response was obtained after antigen targeting to the DEC205^+^ DCs using DNA vaccines consisting of plasmids encoding a single chain variable fragment (scFv) fused to the antigen of interest (36–41). However, other groups have shown that antigen targeting to DEC205^+^ DCs using DNA vaccines was also able to induce immune tolerance to the antigen of interest and consequently protect against experimental allergic encephalomyelitis (42).

In this work, we generated a DNA vaccine encoding the anti-DEC205 scFv fused to DENV2 EDIII (pscDEC-EDIII). Vaccine administration by intramuscular immunization followed by electroporation elicited high titers of anti-EDIII antibodies in mice immunized with pscDEC-EDIII capable of blocking DENV2 infection in VERO cells. In addition, EDIII targeting to DEC205^+^ DCs within the context of a DNA vaccine elicited specific CD4^+^ T cell proliferation and pro-inflammatory cytokine production.

## Materials and Methods

### Plasmid Generation

Constructions of the pcDNA3 scDEC-OVA and scISO-OVA plasmids were described previously (37). We digested the DNA fragment encoding ovalbumin (OVA) with the restriction enzymes *Not*I and *Xba*I (New England Biolabs) and purified the open vectors with the “PureLink Quick Plasmid DNA” kit (Invitrogen). The sequence corresponding to the ectodomain of the DENV2 envelope protein (HQ026763, lineage DENV-2/BR0690/RJ/2008) was synthetized and cloned into the pUC57 plasmid (Genscript USA Inc.). We amplified the EDIII sequence (aa 297–394) with the primers sense 5′ GGCGGCCGCATGTCCTACTCTATGTGCAC 3′ and anti-sense 5′ TCTAGATCAGTGATGGTGATGGTGATGTTTCTTAAACCAATTCAGCTTC 3′ with the Phusion High Fidelity DNA Polymerase (New England Biolabs) according to the manufacturer's instructions. The anti-sense primer was also designed to insert a 6 × His-tag at the end of the sequence. The PCR product was cloned into the pJET1.2/blunt vector (Thermo Scientific) and digested with the restriction enzymes *Not*I and *Xba*I (New England Biolabs). The digestion product was purified with the “PureLink Quick Plasmid DNA” kit (Invitrogen) and cloned in frame with the open reading frames of scDEC and scISO in the pcDNA3 vectors using the T4 DNA ligase enzyme (New England Biolabs). The final vectors, named pscDEC-EDIII and pscISO-EDIII were sequenced to confirm the presence of the EDIII sequence in frame with the antibody sequences. We transformed the plasmids into DH5α bacteria and purified the DNA in large scale using the “EndoFree Plasmid Maxi Kit” (QIAGEN) for subsequently transfection of human embryonic kidney (HEK) 293T cells and mice immunization.

### HEK 293T Transient Transfection

The transfection of HEK293T cells was performed as described previously (30). After 5 days in culture, the supernatants of cultures transfected with pscDEC-EDIII or pscISO-EDIII were collected, centrifuged at 1,000 × g for 30 min and filtered in 0.22 μM filters (Corning). The samples were concentrated in a dialysis membrane surrounded by sucrose and dialyzed twice in PBS for 4 h at 4°C.

### Western Blotting

The scDEC-EDIII or scISO-EDIII containing samples were sorted in a 12% SDS-PAGE gel under reducing conditions. The proteins were transferred to a nitrocellulose membrane (GE Healthcare) and the membrane was blocked overnight at 4°C with PBS containing 0.05% Tween 20 (PBS-T 0.05%), 2.5% BSA and 5% non-fat milk. After three 5-min washes with PBS-T 0.05%, the membrane was incubated with 6x-HIS tag monoclonal antibody (1:5,000; Thermo Fisher Scientific) at room temperature (rt) for 2 h. Next, the membrane was incubated with goat anti-mouse total IgG-HRP antibody (1:2,000; Jackson ImmunoResearch Laboratories) for 2 h at rt and developed using quimioluminescence (ECL kit, GE Healthcare).

### CHO-DEC Binding Assay

Transgenic CHO cells stably expressing the mouse DEC205 receptor (kindly provided by Dr. Michel Nussenzweig, The Rockefeller University, New York) were used for the binding assays. One hundred thousand cells were incubated with 4, 2, or 1 μg/mL of the scDEC-EDIII or scISO-EDIII scFvs for 40 min on ice. After two washes with FACS buffer (2% fetal bovine serum in PBS), cells were incubated with the 6x-HIS tag monoclonal antibody (1:5,000; Thermo Fisher Scientific) for 20 min on ice. The cells were washed two times again with FACS buffer and incubated with the anti-mouse IgG-Alexa488 antibody (1:2,000; Life technologies). After another round of washes, the cells were analyzed by flow cytometry and 20,000 events were acquired in the FACSCalibur™ flow cytometer (BD Biosciences).

### Mice and Immunization

Six- to eight-weeks-old male BALB/c mice were bred at the Isogenic Mouse Facility of the Parasitology Department, University of São Paulo, Brazil. This study was carried out in accordance with the recommendations of the Federal Law 11.794 (2008), the Guide for the Care and Use of Laboratory Animals of the Brazilian National Council of Animal Experimentation (CONCEA) and the ARRIVE guidelines. The Institutional Animal Care and Use Committee (IACUC) of the University of São Paulo approved the protocol under the following number: 36/2016. Groups of eight animals were immunized with 100 μg of pscDEC-EDIII or pscISO-EDIII diluted in saline (0.9% NaCl). A control group consisting of 4 animals was injected with saline alone. Briefly, the animals were anesthetized by intraperitoneal (i.p) injection of a mixture of Ketamine and Xylazine (100 and 10 mg/kg, respectively). Next, the skin over the hind leg was sterilized with ethanol and the injections were carried out intramuscularly (i.m.) in the anterior tibial muscle of the mice followed immediately by electroporation. For the electroporation, two 130 V pulses with 1 ms duration and four 70 V pulses with 50 ms duration were applied with the CUY560-5-0.5 electrode using the NEPA21 Super Electroporator (Nepa Gene Co., Ltd.). The interval between each pulse was 450 ms. Three doses were administered in 2-week intervals. Animals were euthanized 2 weeks after the last dose, their sera were collected via cardiac puncture and the spleens were removed for subsequent analysis.

### ELISA

We used sera collected from the different immunization groups for the detection of EDIII-specific antibodies by ELISA. High binding ELISA plates (Costar) were coated overnight at rt with 100 ng/well of the recombinant EDIII protein (43) diluted in PBS. In the following day, the plates were washed three times with PBS containing 0.02% Tween 20 (PBS-T 0.02%) and then blocked with PBS-T 0.02%, 1% BSA, and 5% non-fat milk for 1 h at rt. After three washes, serum samples were serially diluted in PBS-T 0.02%, 0.25% BSA, and 5% non-fat milk and incubated for 2 h at rt. Goat anti-mouse IgG antibody conjugated with horseradish peroxidase (HRP) (1:2,000; Jackson ImmunoResearch Laboratories) or HRP conjugated subclass-specific anti-mouse IgG (1:2,000; SouthernBiotech) were used as secondary antibodies, and plates were incubated for 2 h at rt. ELISA was developed with ortho-phenylenediaminedihydrochloride (Sigma) and H_2_O_2_ diluted in phosphate–citrate buffer, pH 4.7. The reaction was stopped with 4N H_2_SO_4_ and the OD_490_ was measured in a microplate reader (Biotek). Titers represent the highest serum dilution showing an OD_490_ > 0.1 normalized in a log10 scale. The IgG1/IgG2a ratio was calculated by dividing the mean values of the highest serum dilution obtained for IgG1 by the mean value of the highest serum dilution obtained for IgG2a without normalization. To determine the avidity of the antibodies, we performed an extra step before adding the secondary antibody. Fixed dilutions (OD_490_ = 0.7) of the samples were incubated with 7 M urea or PBS for 5 min. After three washes, the procedure continued exactly as described for the standard ELISA protocol. The avidity index was calculated by the sample's OD_490_ × 100 in 7 M urea divided by the OD_490_ in PBS.

### Virus Neutralization Assay and Competition Assays

Viral neutralization was assessed via a flow cytometry based assay adapted from (44). VERO cells (1 × 10^5^ cells/well) were cultured in flat-bottomed 96-well plates (Costar) overnight at 37°C and 5% CO_2_. Sera from immunized mice were heat inactivated at 56°C for 30 min. Two-fold serially diluted sera were incubated with virus particles of the DENV2 NGC strain (MOI of 0.1) for 30 min at 37°C and 5% CO_2_. The sera/virus mixtures were then incubated with the cells for 1 h at 37°C and 5% CO_2_. Next, the sera/virus mixtures were removed and DMEM containing 5% fetal bovine serum (FBS) was added to the cells that were then incubated for 24 h at 37°C and 5% CO_2_. Trypsin was used to detach the cells that were resuspended in DMEM 5% FBS and transferred to V-bottomed 96-well plates. Cells were fixed and stained as described previously (45) using 4G2 (10 μg/mL; mouse anti-flavivirus envelope antibody) as a primary antibody and anti-mouse IgG-Alexa488 (1:2,000; Life technologies) as a secondary antibody. The cells were resuspended in FACS buffer and 20,000 events were acquired in the BD FACSCalibur™ flow cytometer (BD biosciences). The sera effect on virus neutralization was determined in comparison to a control infection with sera derived from mice injected with saline only.

For the competition assay, adapted from (43), a fixed dilution of the sera from the groups (1:20) was incubated with different molar concentrations of the recombinant EDIII protein for 30 min. The sera/protein mixture was then incubated with the DENV2 NGC strain for 30 min and the experiment continued as described above. Neutralization of infection was determined in comparison to a control infection with DENV2 incubated with recombinant EDIII plus sera from saline injected mice.

### Imunofluorescence

VERO cells (25 × 10^3^ cells/well) were cultured on glass coverslips inside 24-well plates (Costar) overnight at 37°C and 5% CO_2_. Infections were performed with MOI of 0.1 with the DENV2 NGC strain for 1 h. After this period, supernatants containing the virus were discarded and the cells were incubated for 24 h in DMEM 5% FBS. Cells were fixed with ice-cold methanol for 5 min and the coverslips were blocked in PBS containing 1% BSA for 1 h at rt. Next, cells were incubated with pooled sera from the different mouse groups, or with the 4G2 mAb (as a positive control), during 1 h at rt, followed by another incubation with anti-mouse IgG-Alexa488 (1:2,000; Life technologies) in the same conditions. Nuclei were labeled with DAPI (1 μg/mL) and the images were acquired in a fluorescence microscope (Leica DMI6000B/AF6000, Buffalo Grove, IL, USA) coupled to a digital camera system (DFC 365 FX, Leica) and processed by the Leica Application Suite X (LAS X).

### Splenocyte Isolation

Two weeks after the last vaccine dose, spleens were removed aseptically and processed exactly as previously described (30). Pools (*n* = 4; two pools per group) of bulk splenocytes were resuspended in R10 [RPMI supplemented with 10% of FBS (GIBCO), 2 mM L-glutamine (GIBCO), 10 mM Hepes (GIBCO), 1 mM sodium pyruvate (GIBCO), 1% vol/vol non-essential aminoacid solution (GIBCO), 1% vol/vol vitamin solution (GIBCO), 20 μg/mL of ciprobacter (Isofarma, Brazil) and 5 × 10^−5^ M 2-mercaptoetanol (GIBCO)]. Cell viability and concentration were estimated using the Countess™ Automated Cell Counter (Invitrogen).

### Peptide Library

A peptide library comprising the DENV 2 E protein (HQ026763, lineage DENV-2/BR0690/RJ/2008) amino acids 161–404 was synthesized by GenScript USA Inc. This library contained 29 overlapping 20-mer peptides that were synthesized with more than 75% purity. Peptides were resuspended in water (10 mg/mL) and stored at −20°C. For *in vitro* stimulation experiments, peptides were divided into 3 pools as depicted in Table 1.

**Table 1:** List of peptides derived from the E protein.

**Peptide position**	Peptide sequence*	**Domain**	**Pool number**
161–180	EIKITPQSSTTEAELTGYGT	EDI + EDII	1
169–188	STTEAELTGYGTVTMECSPR	EDI + EDII	1
177–196	GYGTVTMECSPRTGLDFNEM	EDI + EDII	1
185–204	CSPRTGLDFNEMVLLQMEDK	EDI + EDII	1
193–212	FNEMVLLQMEDKAWLVHRQW	EDI + EDII	1
201–220	MEDKAWLVHRQWFLDLPLPW	EDI + EDII	1
209–228	HRQWFLDLPLPWLPGADTQG	EDI + EDII	1
217–236	PLPWLPGADTQGSNWIQKET	EDI + EDII	1
225–244	DTQGSNWIQKETLVTFKNPH	EDI + EDII	1
233–252	QKETLVTFKNPHAKKQDVVV	EDI + EDII	1
241–260	KNPHAKKQDVVVLGSQEGAM	EDI + EDII	2
249–268	DVVVLGSQEGAMHTALTGAT	EDI + EDII	2
257–276	EGAMHTALTGATEIQMSSGN	EDI + EDII	2
265–284	TGATEIQMSSGNLLFTGHLK	EDI + EDII	2
273–292	SSGNLLFTGHLKCRLRMDKL	EDI + EDII	2
281–300	GHLKCRLRMDKLQLKG**MSYS**	EDI + EDII + EDIII	2
289–308	**MDKLQLKGMSYSMCTGKFKI**	EDIII	2
297–316	**MSYSMCTGKFKIVKEIAETQ**	EDIII	2
305–324	**KFKIVKEIAETQHGTIVIRV**	EDIII	2
313–332	**AETQHGTIVIRVQYEGDGSP**	EDIII	2
321–340	**VIRVQYEGDGSPCKIPFEIT**	EDIII	3
329–348	**DGSPCKIPFEITDLEKRHVL**	EDIII	3
337–356	**FEITDLEKRHVLGRLITVNP**	EDIII	3
345–364	**RHVLGRLITVNPIVTEKDSP**	EDIII	3
353–372	**TVNPIVTEKDSPVNIEAEPP**	EDIII	3
361–380	**KDSPVNIEAEPPFGDSYIIV**	EDIII	3
369–388	**AEPPFGDSYIIVGVEPGQLK**	EDIII	3
377–396	**YIIVGVEPGQLKLNWFKKGS**	EDIII	3
385–404	**GQLKLNWFKKGSSIGQMF**	EDIII	3

* *Bold shows the EDIII amino acid sequence*.

### ELISpot

We used the mouse IFN gamma ELISPOT Ready-SET-Go!^®;^ (eBioscience) to detect IFN-γ producing splenocytes. The procedure was performed according to the manufacturer‘s instructions. Briefly, ELISpot plates (MAIPS4510; Millipore) were coated with the capture antibody and incubated overnight at 4°C. The plates were washed twice with PBS and blocked for 1 h with R10 at rt. Splenocytes were incubated in the presence of 2 μg/mL pooled EDIII or EDI/II (negative control) peptides for 20 h at 37°C with 5% CO_2_. Unpulsed cells were used as controls for each group. After incubation, the plates were washed three times with PBS-T 0.05% and incubated for 2 h at rt with the biotinylated anti-IFN-γ antibody. Following another round of washes, the plates were incubated with avidin-HRP for 45 min at rt. Plates were washed three times with PBS-T 0.05% and the spots were developed with the “AEC substrate set” kit (BD biosciences). We used an automated stereomicroscope (KS ELISPOT, Zeiss, Oberkochem, Germany) to count the number of spots. The formula (# of spots in the pulsed well – # of spots in the unpulsed well) was used to calculate the number of IFN-γ producing cells/10^6^ cells.

### Proliferation and Intracellular Staining (ICS)

To analyze T cell proliferation, splenocytes from mice were labeled with carboxyfluoresceinsuccinimidyl ester (CFSE). Briefly, 50 × 10^6^ splenocytes were resuspended in pre-heated PBS and labeled with 1.25 μM of CFSE for 10 min at 37°C. Cells were then washed, resuspended in R10 and 3 × 10^5^ cells/well were incubated at 37°C and 5% CO_2_ in 96-well round-bottomed plates with 2 μg/mL of pooled EDIII or EDI/II (negative control) peptides. Unpulsed cells were used as controls for each group. After 3 days in culture, cells were restimulated with the same peptide pools plus 2 μg/mL of αCD28. After 1 h incubation at 37°C and 5% CO_2_, 0.5 μg of Golgi Plug (Brefeldin A, BD Pharmingen) was added per well, and the plate was incubated for 12 h at 37°C and 5% CO_2_. After the incubation period, the cells were washed with FACS buffer and transferred to V-bottomed 96-well plates. Cells were stained with LIVE/DEAD^®^ dye (Life Technologies) and αCD4-PerCP (clone RM4-5) for 40 min on ice and in the dark. After 4 washes with FACS Buffer, the cells were fixed and permeabilized using the Cytofix/Cytoperm kit (BD Pharmingen) according to the manufacturer's instructions. The cells were then washed three times with PermWash buffer (BD Pharmingen). The intracellular staining was performed using αCD3-APC/Cy7 (clone 145-2C11), αIFNγ-APC (clone XMG1.2), αIL2-PE (clone JES6-5H4), and αTNFα-PE/Cy7 (clone MP6-XT22) for 40 min on ice and in the dark. The cells were washed twice and resuspended in FACS Buffer. All antibodies were purchased from BD Pharmingen. Flow cytometer readings were carried out with 200,000 events acquired in the BD LSRFortessa flow cytometer (BD Biosciences) and analyzed using FlowJo software (version 9.3, Tree Star, San Carlo, CA). The analysis of proliferating (CFSE^low^) cells producing different combinations of cytokines (IFNγ, IL-2, and TNFα) was performed with the Boolean gating platform (FlowJo Software). The percentages of proliferating or/and cytokine-producing cells were calculated by subtracting the values obtained with unpulsed cells.

### Data Analysis

We used the Prism 7 software (GraphPad Software Inc, LA Jolla, CA) for all tests. Statistical differences were considered significant when *p* ≤ 0.05. One-way ANOVA followed by Tukey's honestly significantly different (HSD) were used for the ELISA data and Two-way ANOVA followed by Bonferroni correction was used for the ELISpot, CFSE and ICS data. The NT_50_ values for the neutralization assays were determined with the non-linear regression (curve fit) analysis.

## Results

### Production of the Recombinant scFvs

**Figure 1:**
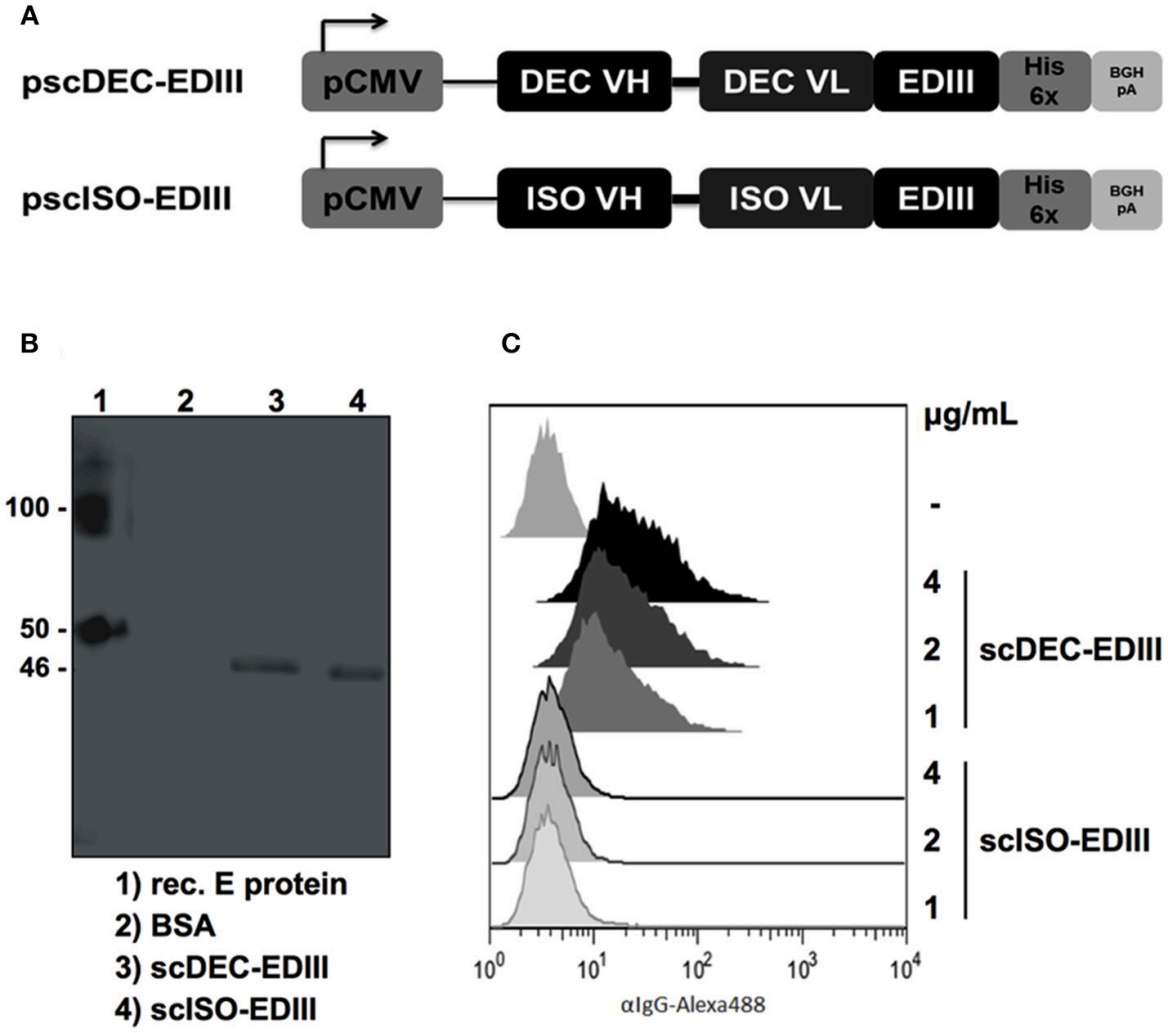
Construction and characterization of the plasmids encoding the EDIII antigen genetically fused with scFvs. **(A)** Map of the plasmid vectors encoding the scFvs fused to the EDIII antigen. The EDIII DNA sequence was cloned in frame with the C-terminal portion of the variable light-chain (VL) after the linker sequence GGSSGGSGGGGSGGGGR. The variable heavy-chain (VH) is connected to the VL via a linker (GGGGS)_3_. pCMV: Cytomegalovirus promoter; His 6x: polyhistidine tag; BGH pA: bovine growth hormone polyadenylation signal. **(B)** Western blotting with the supernatant of HEK293T cells transfected with pscDEC-EDIII and pscISO-EDIII. The membrane was incubated with a 6x-HIS tag mAb followed by incubation with goat anti-mouse total IgG-HRP. E protein: DENV2 envelope protein; BSA: bovine serum albumin. Numbers on the side indicate the molecular weight (kDa). **(C)** Binding of scDEC-EDIII or scISO-EDIII to the DEC205 receptor constitutively expressed by CHO cells. One hundred thousand CHO cells were incubated with 4, 2 or 1 μg/mL of either scFv. Cells were labeled with a 6x-HIS tag mAb followed by incubation with goat anti-mouse IgG-Alexa488. Analysis was performed using the FlowJo software (version 9.3, Tree Star).

The DENV2 EDIII nucleotide sequence (encoding amino acids 297–394) was cloned in frame into plasmids encoding the variable regions of the heavy and light chains of the anti-DEC205 (clone NLDC145) and the isotype control (clone III/10) as previously described (37). Figure 1A shows a schematic representation of pscDEC-EDIII and pscISO-EDIII that were then used to transfect HEK293T cells. Western blot analyses of concentrated cell culture supernatants confirmed secretion of scDEC-EDIII and scISO-EDIII by transfected cells (~46 kDa, Figure 1B). To demonstrate that scDEC-EDIII retained the capacity to bind to the DEC205 receptor, CHO cells stably expressing the murine DEC205 receptor were incubated with different concentrations of either scDEC-EDIII or scISO-EDIII. Figure 1C shows that only the scDEC-EDIII bound to DEC205 receptor in a concentration dependent manner. Taken together, these results indicate that both scFvs were successfully secreted from transiently transfected cells, and that the scDEC-EDIII preserved its binding capacity to the DEC205 receptor.

### *In vivo* EDIII Targeting to DCs Improves Antibody Responses

**Figure 2:**
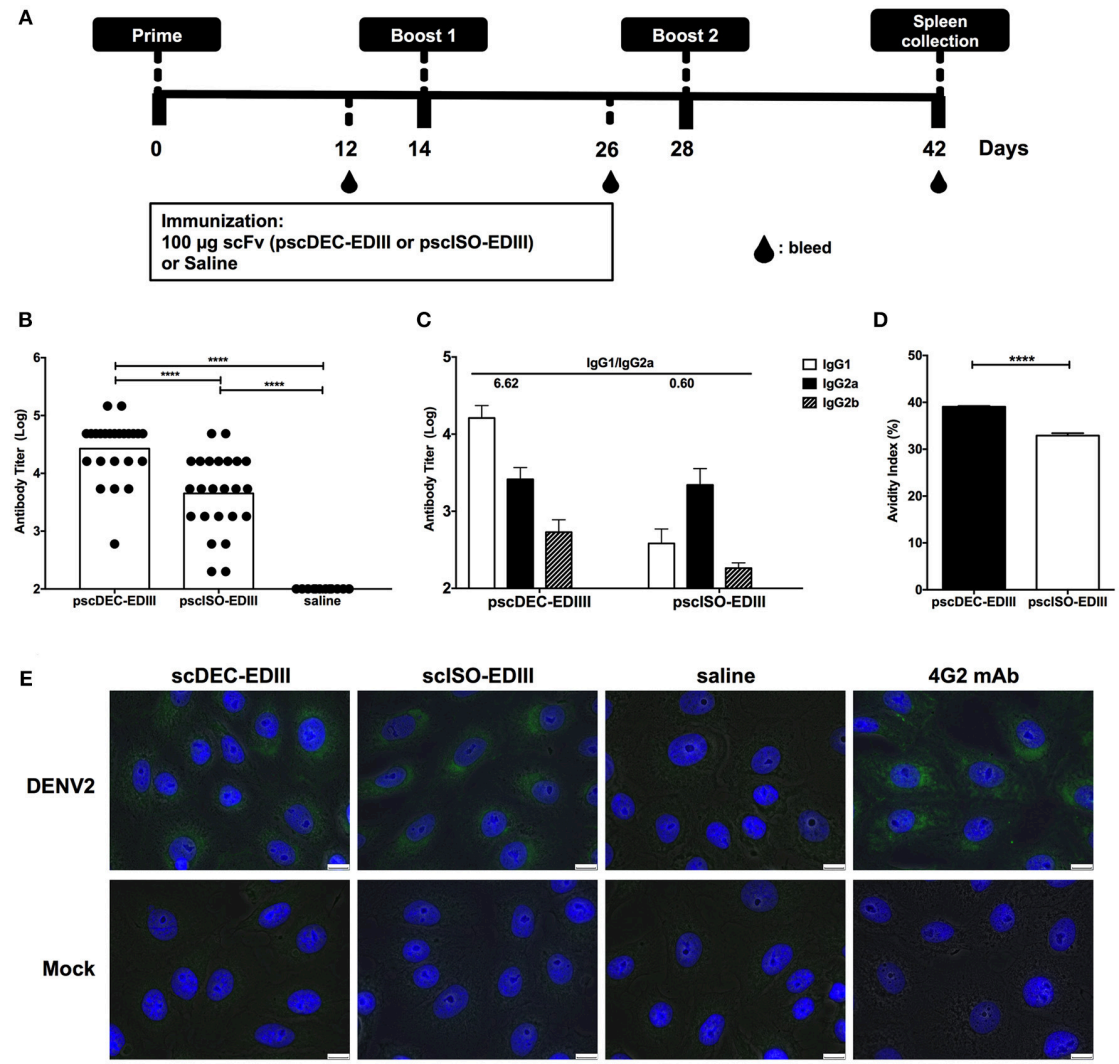
Anti-EDIII antibody responses in mice immunized with pscDEC-EDIII and pscISO-EDIII. **(A)** Immunization regimen. Groups of BALB/c mice were immunized i.m. with the plasmids followed by electroporation. Three doses were given in 2-weeks intervals. **(B)** Total anti-EDIII IgG antibody titers and **(C)** IgG subclasses, 14 days after the administration of the third vaccine dose (*n* = 24; pscDEC-EDIII and pscISO-EDIII and *n* = 12; Saline, from three independent experiments). ELISAs were performed using recombinant EDIII as antigen and developed using a goat anti-mouse total or subclass specific IgG-HRP antibodies. Graphs show the antibody titers in normalized log10 scale. **(D)** Avidity of the anti-EDIII antibodies. Fixed dilutions of the pooled group samples were incubated with 7 M urea or PBS. The avidity index was calculated by the sample's OD_490_ × 100 in 7M urea divided by the OD_490_ in PBS. Symbols represent individual mice, columns, and bars represent the mean and SD for each group. Data were analyzed by a one-way ANOVA followed by the post-test HSD Tukey **(B)** or by unpaired *t*-test **(D)**. *P*-value indicators **** refer to *p* < 0.0001. **(E)** Recognition of the DENV2 virus by the sera from mice immunized with pscDEC-EDIII and pscISO-EDIII. VERO cells were infected with DENV2 NGC strain or mock (medium). Cells were fixed with methanol and incubated with the sera from mice immunized with either scFv. The virus/sera and nuclei were labeled with the anti-mouse IgG-Alexa488 (green) and DAPI (blue), respectively. The 4G2 mAb was used as a control. Results shown are representative of two independent experiments. Scale bar, 10 μm.

Next, we assessed if immunization with pscDEC-EDIII could improve the anti-EDIII antibody response. For that purpose, mice received three doses of each plasmid administered i.m. followed by electroporation (Figure 2A). Twelve days after the administration of the first and second doses, and 14 days after the third dose, mice were bled and the sera were tested individually for reactivity against the recombinant EDIII produced in bacteria. Supplementary Figure 1 shows an increase in anti-EDIII antibody titers in mice immunized with both plasmids, especially after the administration of the third dose. Moreover, the anti-EDIII antibody titers observed in the animals immunized the pscDEC-EDIII were higher than those observed in mice immunized with pscISO-EDIII (Figure 2B). When IgG subclasses present in the sera of immunized mice were analyzed, IgG1, IgG2a, and IgG2b but not IgG3 were detected (Figure 2C). Notably, the IgG1/IgG2a ratio detected in mice immunized with pscDEC-EDIII was approximately 10 × higher (6.62) than the one obtained in mice immunized with pscISO-EDIII (0.60). When antibody avidity was measured, we noticed that it was higher in sera from mice immunized with pscDEC-EDIII than in mice immunized with pscISO-EDIII (Figure 2D). Anti-EDIII antibodies were also tested for binding to the viral E protein by immunofluorescence and, as expected, sera collected from mice immunized with pscDEC-EDIII or pscISO-EDIII reacted with VERO cells previously infected with DENV2 (Figure 2E). The labeling patterns were similar to those observed in infected cells stained with 4G2 mAb that recognizes the E protein of Flaviviruses. These results indicate that there are differences in the magnitude, and in the quality of anti-EDIII antibodies raised after immunization with pscDEC-EDIII or pscISO-EDIII.

### Anti-EDIII Antibodies Raised in Immunized Mice Inhibit DENV2 Infection

**Figure 3:**
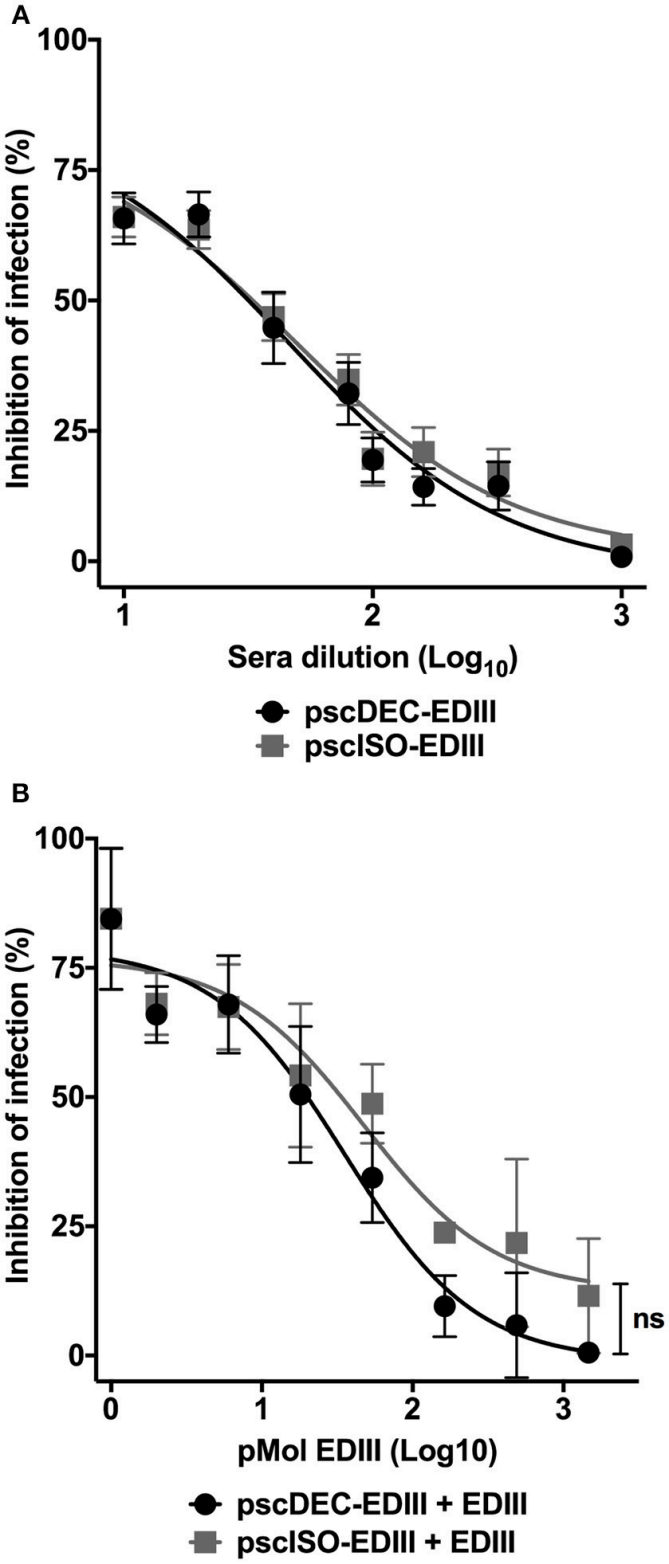
Antibodies from mice immunized with pscDEC-EDIII and pscISO-EDIII partially inhibit DENV infection by binding to EDIII. **(A)** Neutralization in VERO cells. Pooled sera from mice immunized as described in Figure 2 were heat inactivated at 56°C for 30 min. Two-fold serially diluted sera were incubated with the DENV2 particles for 30 min at 37°C and 5% CO_2_. The sera/virus mixture was then incubated with the cells for 1 h at 37°C and 5% CO_2_. The cultures supernatant was replaced by DMEM 5% FBS followed by another incubation at the same conditions for 24 h. Cells were stained with the 4G2 (mouse anti-flavivirus envelope antibody) and anti-mouse IgG-Alexa488. The neutralization of infection was determined in comparison to a control infection. Results are represented by means and SEM from pooled data of four independent experiments. **(B)** Competition assay with the recombinant EDIII protein. A fixed dilution (1:20) of sera was incubated with increasing molar concentrations of EDIII protein prior to incubation with the virus. The cells were resuspended in FACS buffer and 20,000 events were acquired in the BD FACSCalibur™ (BD biosciences) flow cytometer. The neutralization of infection was determined in comparison to a control infection with sera derived from mice injected with saline. Data were analyzed by a two-way ANOVA for repetitive measures followed by Sidak's multiple comparisons test. ns = not-significant. Representative of three independent experiments.

Anti-EDIII antibodies were shown to be effective to block virus entry into eukaryotic cells (7–9, 46, 47). We then analyzed if anti-EDIII antibodies present in the sera of immunized mice were able to block DENV2 infection. Figure 3A shows that antibodies raised in mice immunized with both scFvs plasmids inhibited virus entry with the same efficiency and in a dilution dependent manner. The 50% neutralization titers (NT_50_) were also similar for sera derived from pscDEC-EDIII (1.66) or from pscISO-EDIII (1.69) immunized mice. To verify if the anti-EDIII antibodies were able to block DENV2 infectivity by binding to EDIII, we performed a competition assay using different concentrations of recombinant EDIII and a fixed serum dilution. Sera derived from mice immunized with pscDEC-EDIII, or pscISO-EDIII were then incubated with increasing amounts of EDIII. We observed a reduction in the sera capacity to inhibit DENV infection as the amount of EDIII was increased (Figure 3B). Of note, although we noticed a difference in the slope of the curves between the two groups, no statistical significance was observed, indicating that the anti-EDIII antibodies induced in both groups bound equally well to recombinant EDIII.

These results indicate that immunization with a DC targeted DNA vaccine was able to elicit higher anti-EDIII antibody titers with higher avidity. However, their blocking capacity did not differ from the blocking capacity of anti-EDIII antibodies induced by the vaccine that did not target DCs.

### DC-Targeted DNA Vaccine Elicits Specific IFNγ Production and CD4^+^ T Cell Proliferation

**Figure 4:**
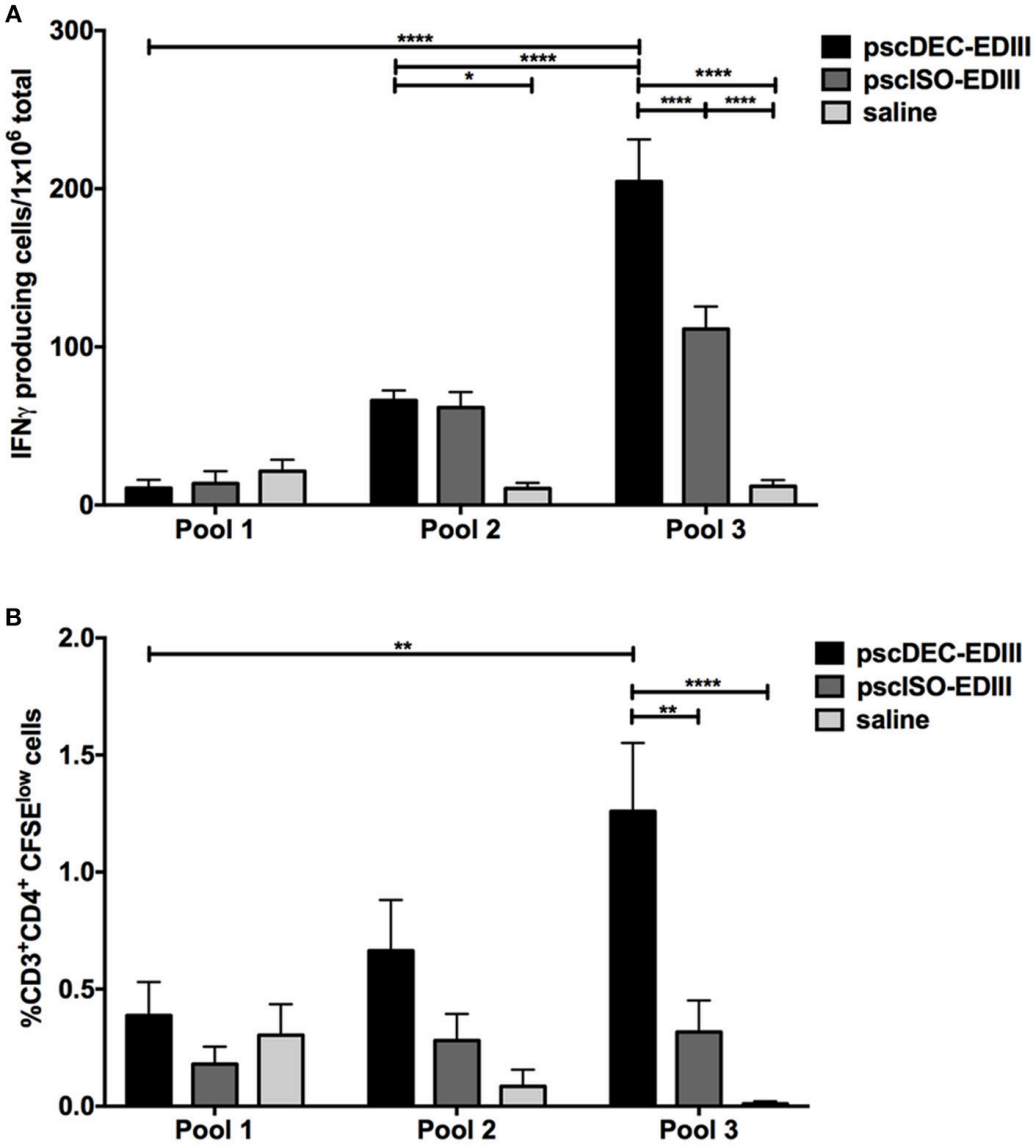
Vaccination with pscDEC-EDIII induces IFNγ production and CD4^+^ T cell proliferation. Groups of mice were immunized as described in Figure 2. **(A)** IFNγ ELISpot. Splenocytes were stimulated with 2 μg/mL of the peptides pools (Table 1). **(B)** Proliferation of CD3^+^CD4^+^ cells measured after CFSE staining. Splenocytes were stained with CFSE and stimulated with the same peptide pools (Table 1). After 3 days in culture, cells were pulsed again with the pools and with the αCD28 mAb. The frequency of cells that lost CFSE was determined by flow cytometry on the 4th day. The gating strategy is shown in Supplementary Figure 2. Columns and bars in both graphs represent the mean and SEM of pooled data from three independent experiments. The amount of spots/proliferation was determined after subtraction of values obtained in non-stimulated samples. Data were analyzed by a two-way ANOVA followed by the post-test HSD Tukey. *P*-value indicators *, ** and **** refer to *p* < 0.05, *p* < 0.01, and *p* < 0.0001, respectively.

We also investigated if EDIII targeting to DCs would impact cellular immune responses, particularly CD4^+^ T cells. Splenocytes harvested 14 days after the administration of the last immunization dose were incubated with three peptide pools containing peptides derived from a library comprising EDI/II and EDIII 20-mer overlapping peptides (Table 1). Pool 1 contained 10 peptides restricted to EDI/II domains (not present in the EDIII sequence encoded by the DNA vaccine plasmids), pool 2 contained five peptides derived from EDI/II and five peptides derived from EDIII sequence, and pool 3 contained nine peptides derived from EDIII amino acid sequence. All three pools were used to stimulate splenocytes from mice immunized with pscDEC-EDIII, pscISO-EDIII or saline. ELISpot assays showed that the number of IFNγ-producing splenocytes derived from mice immunized with pscDEC-EDIII was higher than that observed in mice immunized with the isotype control DNA vaccine or saline (Figure 4A). In addition, the response was mainly directed to a peptide(s) contained in pool 3 (Figure 4A). There was also a statistically significant difference in the number of IFNγ-expressing cells derived from mice immunized with pscDEC-EDIII after stimulation with peptide pool 2. This result suggests that pools 2 and 3 contain peptides able to bind to the BALB/c H-2K^d^ haplotype. When CD4^+^ T cell proliferation (representative gating strategy shown in Supplementary Figure 2, CFSE^low^ panel) was analyzed, we also detected a higher frequency of CD3^+^CD4^+^CFSE^low^ cells in the DC-targeted DNA vaccine group when compared to the groups immunized with the isotype control plasmid or saline (Figure 4B). Similarly to the previous experiment, the highest frequency of proliferation was directed against pool 3. As expected, pool 1 (comprising unrelated peptides derived from EDI/II domain) elicited a lower response in mice immunized with scFv plasmids that was not different from the response induced in animals that received saline.

### DC-Targeted DNA Vaccine Induces CD4^+^ T Cells That Proliferate and Produce Pro-Inflammatory Cytokines at the Same Time

**Figure 5:**
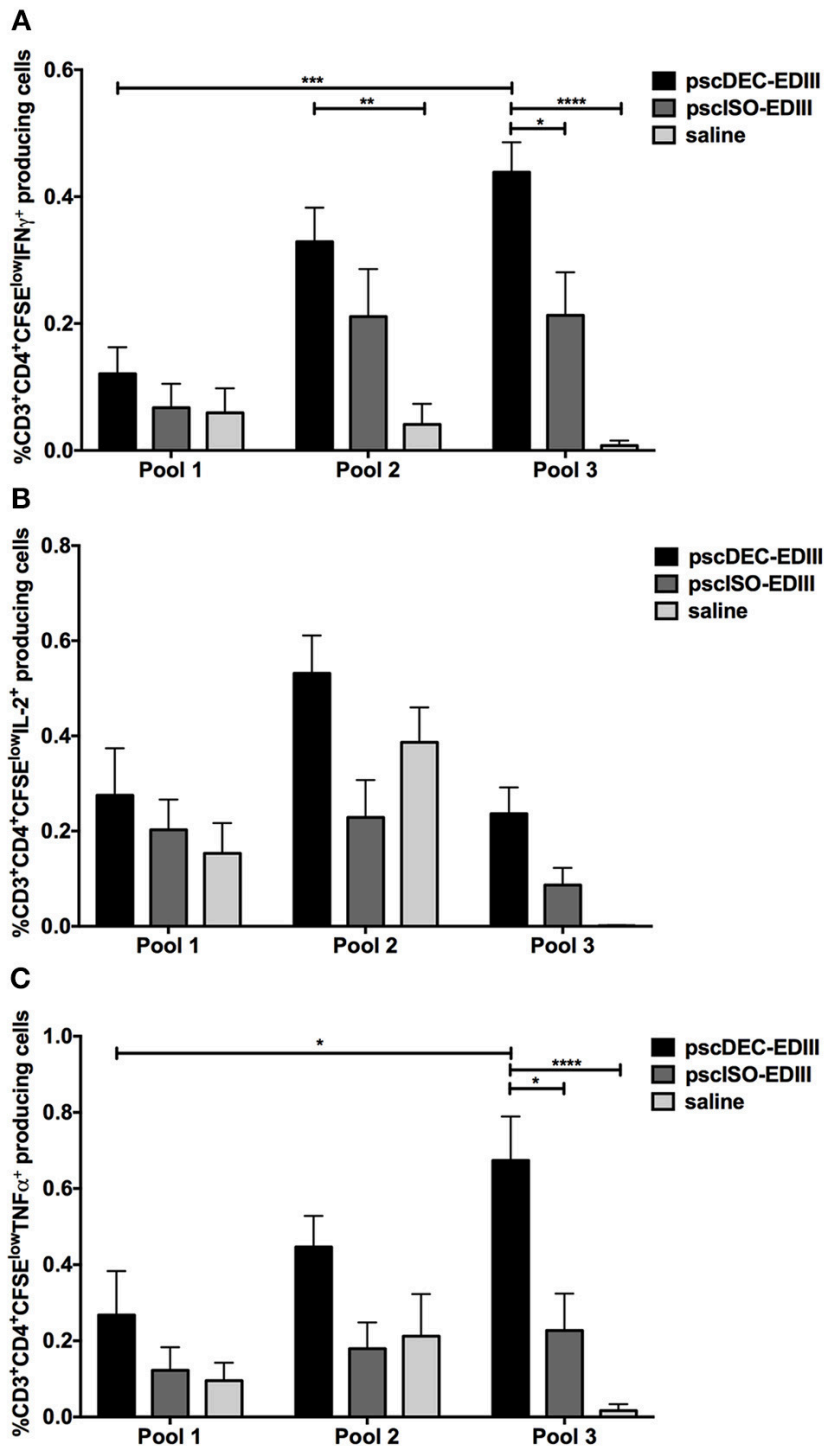
The pscDEC-EDIII vaccine induces pro-inflammatory cytokines in proliferating CD4^+^ T cells. Groups of mice were immunized as described in Figure 2 and cytokine production was evaluated by intracellular cytokine staining. **(A–C)** Splenocytes were stimulated with 2 μg/mL of peptide pools (Table 1). After 3 days in culture, the cells were pulsed again with the pools and with the αCD28 mAb. The frequency of CD3^+^CD4^+^ cells that produced **(A)** IFNγ, **(B)** IL-2 or **(C)** TNFα within the CFSE^low^ population was determined by flow cytometry on the 4th day. The gating strategy is shown in Supplementary Figure 3. Columns and bars in both graphs represent the mean and SEM of pooled data from three independent experiments. The percentage of CFSE^low^ cells producing cytokines was determined after subtraction of values obtained in non-stimulated samples. Data were analyzed by a two-way ANOVA followed by the post-test HSD Tukey. *P*-value indicators *, **, *** and **** refer to *p* < 0.05, *p* < 0.01, *p* < 0.001 and *p* < 0.0001, respectively.

We next assessed if CD4^+^ T cells that proliferated in response to pools 2 and 3 were also able to produce the pro-inflammatory cytokines IFNγ, IL-2, and TNFα (representative gating strategy shown in Supplementary Figure 3). As shown in Figure 5A, the frequency of CD4^+^ T cells that proliferated and produced IFNγ was higher in animals immunized with pscDEC-EDIII pulsed with pools 2 and 3 when compared to animals that received saline. For pool 3, as observed in the ELISpot assay, the frequency of CD4^+^CFSE^low^ that produced IFNγ was also higher in mice immunized with pscDEC-EDIII than in the group immunized with pscISO-EDIII. We did not observe significant differences among the groups when we compared the frequency of CD4^+^CFSE^low^ cells that produced IL-2 (Figure 5B). TNFα production by proliferating CD4^+^ T cells was also higher in cells derived from pscDEC-EDIII immunized mice, especially when they were incubated with pool 3. A higher response against pool 2 was also observed, but it did not reach statistical significance when compared to the other two groups (Figure 5C).

**Figure 6:**
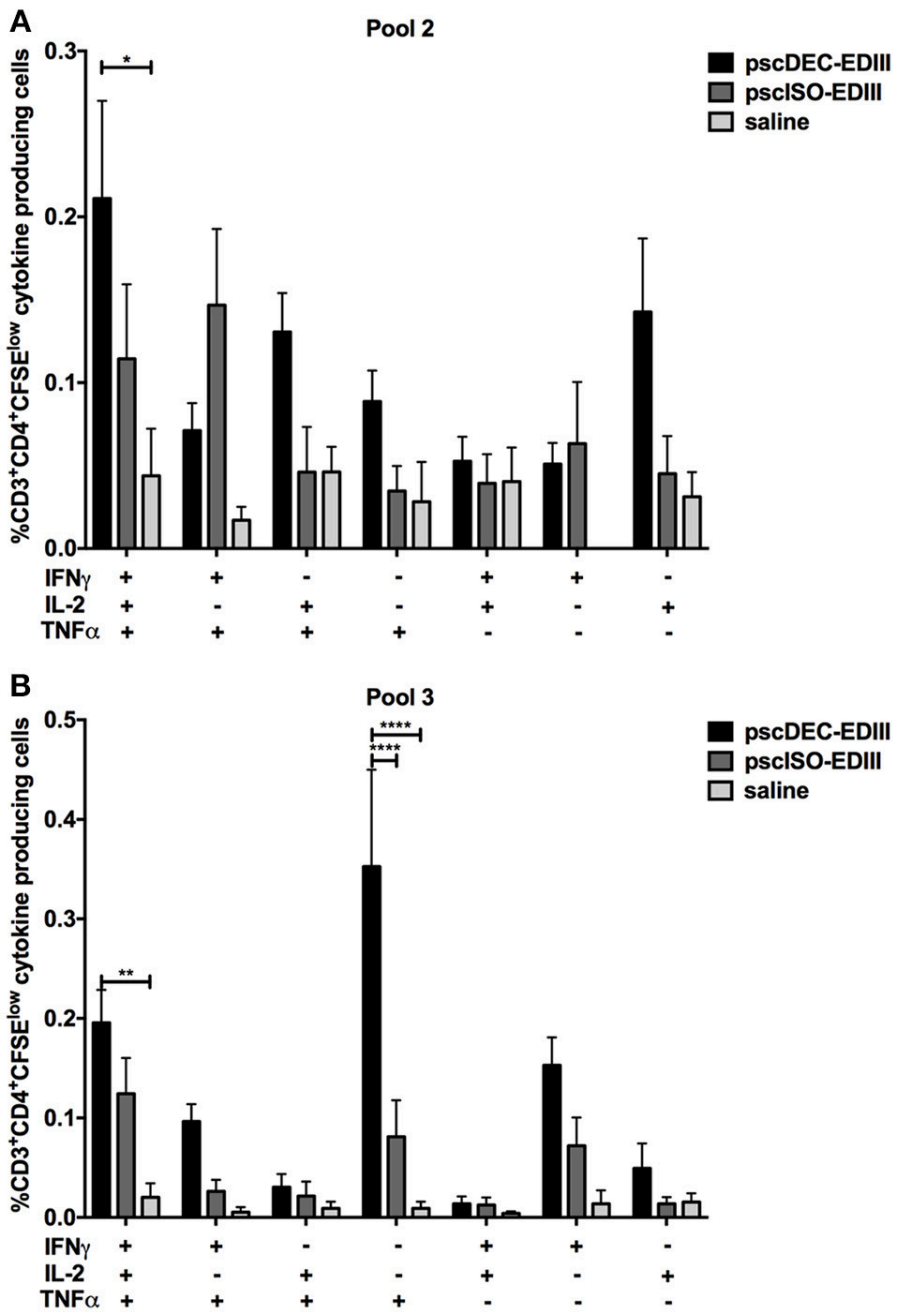
Boolean analysis of the CD3^+^CD4^+^CFSE^low^ cells producing different combinations of IFNγ, IL-2, and TNFα. Groups of mice were immunized as described in Figure 2. Splenocytes were stimulated with 2 μg/mL of peptide pool 2 **(A)** or 3 **(B)**. After 3 days in culture, cells were pulsed again with the peptide pools and αCD28 mAb. Proliferation and IFN**γ**, IL-2, and TNFα production were evaluated by CFSE staining and ICS, respectively. The Boolean gating platform was used to analyze all the different possible combinations of CD3^+^CD4^+^CFSE^low^ cells expressing each or combinations of the cytokines. The gating strategy is shown in Supplementary Figure 2. Columns and bars in both graphs represent the mean and SEM of pooled data from two independent experiments. The frequencies of CFSE^low^ cells producing cytokines were determined after subtraction of values obtained in non-stimulated samples. Data were analyzed by a two-way ANOVA followed by the post-test HSD Tukey. *P*-value indicators *, **, and **** refer to *p* < 0.05, *p* < 0.01 and *p* < 0.0001, respectively.

The results in Figure 5 showed that immunization with pscDEC-EDIII induced CD4^+^ T cells that proliferated and produced three pro-inflammatory cytokines mainly to peptides contained in pools 2 and 3. To explore this response in more detail, we performed a Boolean analysis of the data (representative gating strategy shown in Supplementary Figure 2, CFSE^low^ and cytokine^+^ panels), and showed that the CD4^+^CFSE^low^ cells were polyfunctional and able to produce combinations of the tested cytokines. For example, proliferating CD4^+^ T cells from mice immunized with pscDEC-EDIII produced all three cytokines simultaneously when pulsed with pool 2 (Figure 6A). Despite not statistically significant, we also observed an increase in the frequencies of CD4^+^ T cells that proliferated and produced IL-2/TNFα, or only IL-2 or TNFα in pscDEC-EDIII immunized animals. The only exception was due to the IFNγ/TNFα double positive cells whose frequency was higher in pscISO-EDIII immunized animals. Similar results were observed in assays using pool 3 (Figure 6B). Mice immunized with pscDEC-EDIII showed a statistically significant increase in the frequency of triple positive CD4^+^ T cells when compared to the group that received saline. Moreover, the percentage of CD3^+^CD4^+^ cells that proliferated and produced only TNFα was statistically higher in mice immunized with pscDEC-EDIII than in those that received pscISO-EDIII or saline. Despite not significant, we observed that animals immunized with pscDEC-EDIII presented a higher frequency of cells positive for IFNγ/TNFα, only IFNγ, or only IL-2 when compared with the other two groups. Taken together, these results indicate that EDIII targeting to DCs using a DNA vaccine was able to elicit a polyfunctional CD4^+^ T cell response.

### Identification of EDIII-Specific Epitopes Recognized by CD4^+^ T Cells Elicited in Mice Immunized With pscDEC-EDIII

**Figure 7:**
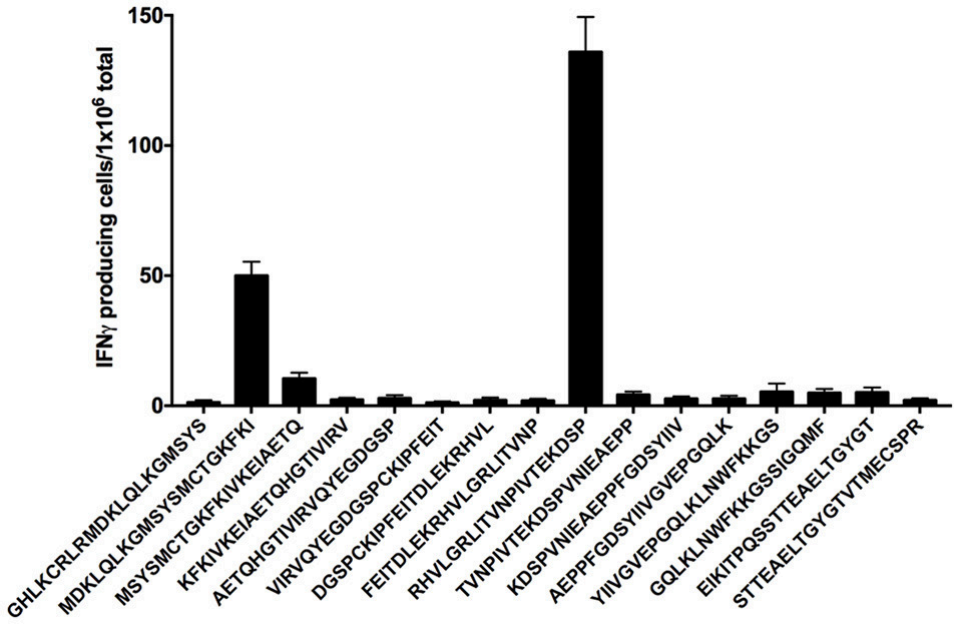
Two peptides from the EDIII sequence induce IFNγ production. Groups of mice were immunized as described in Figure 2 and an IFNγ ELISpot was performed. Splenocytes from mice immunized with pscDEC-EDIII were stimulated with 2 μg/mL of peptides spanning the entire EDIII sequence. Peptides EIKITPQSSTTEAELTGYGT and STTEAELTGYGTVTMECSPR from EDI/II sequence were used as negative controls. Columns and bars represent the mean and SEM of pooled data from two independent experiments. The number of spots was determined after subtraction of values obtained in non-stimulated samples.

In order to identify the peptide(s) present in the pools able to specifically activate CD4^+^ T cell responses in mice immunized with pscDEC-EDIII, we performed an ELISpot assay using individual peptides comprising the complete EDIII sequence plus two control peptides derived from EDI/EDII (EIKITPQSSTTEAELTGYGT and STTEAELTGYGTVTMECSPR). Splenocytes from mice immunized with pscDEC-EDIII were pulsed with each individual peptide and the number of IFNγ producing splenocytes/10^6^ total cells was recorded. Figure 7 indicates that the response was mainly directed to two peptides: MDKLQLKGMSYSMCTGKFKI present in pool 2, and RHVLGRLITVNPIVTEKDSP in pool 3. In addition, we observed that peptide RHVLGRLITVNPIVTEKDSP represented the immunodominant epitope since the response directed to it was almost three times higher than that detected after stimulation with peptide MDKLQLKGMSYSMCTGKFKI.

## Discussion

Dengue infection has become a major public health concern as the disease outbreaks and complications have increased substantially in the last five decades (48). Since then, the development of a vaccine has become a global health priority. The challenge is enormous as dengue is caused by four different serotypes and a previous immune response against one particular serotype can exacerbate the disease caused by another (8). Different approaches are being evaluated and two vaccines based on live attenuated viruses have reached phase III trials: the CYD-TDV by Sanofi Pasteur and the TV003/TV005 by US National Institutes of Health (49). However, results of the CYD-TDV vaccine indicating that the risk of severe disease could increase in seronegative individuals led WHO to recommend that the vaccine would only be administered in populations with dengue serological prevalence rates above 80% (50). In this way, other approaches are currently being developed.

Antigen targeting to DCs through the use of chimeric mAbs has been a promising strategy to induce either humoral or cellular immune responses against different antigens such as: ovalbumin (26–28), *Plasmodium yoelii* circumsporozoite protein (27), *Yersinia pestis* LcrV (29), DENV2 non-structural protein 1 (30), *Trypanosoma cruzi* amastigote surface protein 2 (31), *Plasmodium vivax* merozoite surface protein 1 (32, 33), and HIV gag (51, 52), among others.

However, the production of such mAbs is time consuming and expensive. DNA vaccines, on the other hand, are cheap, safe and easier to produce and purify, but in general are less immunogenic (35). Different approaches have been developed to increase the immunogenicity of DNA vaccines. Among them are the use of electroporation (53, 54) and the use of plasmids encoding scFv specific for the DEC205 receptor coupled to the antigen of interest.

Although EDIII has been recently used in a DNA immunization strategy (9), in this work we sought to produce a DNA vaccine able to target EDIII from DENV2 to the DEC205^+^ DCs *in vivo*. This was accomplished when we fused the sequence of anti-DEC205 scFv to the EDIII, generating the DNA plasmid named pscDEC-EDIII. As a negative control, we also fused the scFv of a control mAb that was not able to bind to DCs (pscISO-EDIII). Interestingly, a similar construct was engineered by Coconi-Linares et al. that expressed a scFvDEC-EDIII in the plant *Nicotiana benthamiana* (55). In this case, the authors purified the recombinant scFvDEC205-EDIII protein and immunized BALB/c mice in the presence of anti-CD40 and poly (I:C). Their results showed that the scFvDEC205-EDIII was immunogenic, inducing antibodies with neutralization capacity and proliferating T cells. Nonetheless, a more detailed evaluation of the antibody and T cell responses was not performed.

We decided to use the pscDEC-EDIII as a DNA vaccine for its simplicity and potential to induce strong immune responses when administered together with electroporation. Initially we showed that both plasmids (pscDEC-EDIII or its isotype control) were able to drive the production of chimeric scFvs when transfected in HEK293T cells. More importantly, the scFvs were successfully secreted from the cells, indicating availability in the extracellular medium and possible targeting to the DEC205^+^ DCs. Indeed, scDEC-EDIII showed a concentration dependent binding to the DEC205 receptor. Similar results were obtained with other antigens coupled to the same scFvs (37, 56).

Once production and DEC205 receptor specific binding were confirmed for scDEC-EDIII, we used scFv plasmids to immunize mice. We showed that the anti-E antibody titers after the administration of three doses were higher in the group immunized with pscDEC-EDIII when compared to the non-targeted control. Others obtained similar results using different antigens like ovalbumin, HIV gag p41 (37), HER2/neu (38), hepatitis B virus (57), human respiratory syncytial virus (40), and botulinum neurotoxin (39). Although the number of doses varied as well as the amount of plasmid DNA, all these studies used electroporation following intramuscular injection. Interestingly, when we analyzed the IgG isotypes elicited by immunization with the scFvs, we noticed that there was a difference in the IgG1/IgG2a ratio in the sera of mice immunized with pscDEC-EDIII or pscISO-EDIII. Although some groups, using different antigens, also obtained similar results (37, 58), others showed differences when both groups were compared (38). Despite the differences from one study to another, it has become clear that antigen targeting to the DEC205^+^ DCs modulates the humoral immune response differently than the non-targeted antigen. We also noticed a significant increase in the avidity of the anti-E antibodies raised in mice immunized with pscDEC-EDIII, while antibodies derived from both immunized groups recognized infected cells. A similar recognition pattern was also obtained after intradermal immunization with a DNA plasmid encoding EDIII (9). This result indicated that the EDIII recognized by the mice sera presented a similar conformation when compared to the EDIII present in the viral particle.

EDIII has been previously described as a target for neutralizing antibodies (46, 47, 59, 60). We then decided to analyze if sera from scFv immunized animals were able to block virus invasion *in vitro*. The results showed that sera from mice immunized with pscDEC-EDIII or with pscISO-EDIII blocked DENV2 infection in a dilution dependent manner. This result contrasted with our previous results showing higher titers and avidity in the sera of mice immunized with pscDEC-EDIII. A positive correlation between neutralization capacity and higher avidity to the viral particle was observed previously on dengue-infected patients (61). In contrast, other results using HIV envelope proteins showed that higher avidity not always correlates with neutralization capacity (62). More importantly, when sera from these mice were previously incubated with different amounts of recombinant EDIII, we observed a reversion in the sera capacity to block infection. This result more clearly demonstrated that the antibodies directed to EDIII mediate DENV infection inhibition in this model. Similar results were also obtained when an EDIII recombinant protein was administered in the presence of the heat-labile toxin (LT) or its non-toxic B subunit (43), or when a chimeric protein containing EDIII was administered to monkeys (63).

Our group and others described that antigen targeting to the DEC205^+^ DCs is a very efficient way of elicit CD4^+^ T cell responses (27, 30–34, 51, 64, 65). We then sought to analyze the CD4^+^ T cell response induced after immunization with plasmids encoding scFvs genetically fused to EDIII. We took advantage of a peptide library comprising overlapping peptides derived from the E protein amino acids 161 to 404. Our data showed that the pscDEC-EDIII immunization induced CD4^+^ T cells that proliferated when pulsed with peptide pools comprising the EDIII portion of the molecule. An analysis of pro-inflammatory cytokines produced by the animals immunized with pscDEC-EDIII showed a higher frequency of IFNγ and TNFα producing CD4^+^ T cells when compared to the animals immunized with the isotype control, although IL-2 levels were comparable. Interestingly, there was a difference in the magnitude of the response when splenocytes were pulsed with pools 2 or 3. The frequency of CD4^+^ T cells that proliferated and produced IFNγ or TNFα was higher after pulse with pool 3, indicating the presence of an immunodominant epitope(s) capable of binding the BALB/c haplotype (H-2K^d^). A more detailed analysis showed that pscDEC-EDIII immunization elicited polyfunctional CD4^+^ T cells producing one, two, or three pro-inflammatory cytokines, even though statistical significance in comparison to the isotype control group was only reached when single TNFα producers were compared. CD4^+^ T cells producing the same combination of pro-inflammatory cytokines were also observed in animals immunized with scDEC-HIV p41 (37) or with scDEC-HER2 (38). The presence of polyfunctional CD4^+^ T cells with protective capacity was first described in a mouse model testing a vaccine against *Leishmania major* (66). After that, many groups set out to investigate if polyfunctional CD4^+^ T cells could be related with protection in other models. Studies with HIV-1 infected individuals showed that those displaying polyfunctional CD4^+^ T cells were able to better control disease (67, 68). In dengue infection, one study showed that CD4^+^ T cells that produced either IFNγ or IL-2 correlated with protection from secondary virus infection in children (69). Polyfunctional CD4^+^ T cells were also identified in individuals submitted to a DENV-1 vaccine candidate, although protection against infection was not investigated in this particular study (70).

Worth mentioning is the fact that, in some cases, DNA vaccination with a scFv encoding the scDEC205 fused with an antigen elicited weaker immune responses when compared to the non-targeted controls (56, 58, 71). The reason for these results is still unclear. In fact, DEC205 targeting using chimeric anti-DEC205 mAbs has been known to induce tolerance if the antigen is delivered in the absence of a DC maturation stimulus (72). However, electroporation facilitates DNA uptake and makes more DNA available for detection by intracellular DNA sensors, thereby activating the production of cytokines as an innate reaction (73). In addition, the plasmid backbone should also be considered. As a mechanistic explanation is still elusive, additional studies are necessary.

Finally, we attempted to map the epitopes responsible for the CD4^+^ T cell proliferation and cytokine production. When splenocytes from mice immunized with pscDEC-EDIII were pulsed with each individual peptide, we identified two peptides that were probably responsible for the response observed against pools 2 and 3: MDKLQLKGMSYSMCTGKFKI and RHVLGRLITVNPIVTEKDSP, respectively. Peptide RHVLGRLITVNPIVTEKDSP comprises the sequence of peptide RHVLGRLITVNPIVT that was shown to induce IFNγ production when this peptide was used to immunize C57BL/6J mice (74). In addition, the same peptide was also shown to bind to HLA-DRB1^*^08:02 in patients from Nicaragua (75) and Sri Lanka (76). This result indicates that our immunization strategy may have the potential to induce CD4^+^ T cells in humans. Peptide MDKLQLKGMSYSMCTGKFKI is contained in the sequence of peptide SGNLLFTGHLKCRLRMDKLQLKGMSYSMCTG, which was previously used to immunize BALB/c mice. Proliferation and release of IL-2 were detected in this case (77). As DEC205 targeting greatly improves CD4^+^ T cell responses, it is relatively easy to map antigenic peptides. CD4^+^ T cell epitopes derived from different proteins have been more easily mapped in samples derived from animals submitted to antigen targeting to DCs. CD4^+^ T cells epitopes were detected in the *Plasmodium yoelii* circunsporozoite protein (27), in the HIV p24 gag (51), in the *Leishmania major* LACK antigen (64), in the *Yersinia pestis* LcrV antigen (65) and in the *Trypanosoma cruzi* ASP-2 protein (31).

The role of CD4^+^ T cells in dengue infection is still not very well defined. CD4^+^ T cells from infected patients were found to have *ex vivo* specific cytolytic activity against DENV (13). Individuals vaccinated with an experimental live attenuated DENV1 vaccine exhibited CD4^+^ T cells with cytotoxic and proliferative capacities *in vitro* (78, 79). In mouse models, a DNA vaccine encoding the NS1 protein induced protection via CD4^+^ T cells and antibodies (80). Immunization with a chimeric αDEC205 mAb fused to NS1 was also able to induce CD4^+^ T cells that contributed for protection (30). Yauch et al. showed that vaccination with CD4^+^ peptides in an IFN-α/βR^−/−^ mouse model reduced viral loads. Interestingly, CD4^+^ T cells did not seem to have an impact on antibody neutralization of the virus measured by infection of C6/36 cells (81). Further studies will be needed to address the role of CD4^+^ T cells in our model.

Although in the literature CD8α^+^ DCs are described as playing an important role in the uptake of apoptotic cells and in antigen cross-presentation in the context of MHC Class I (82, 83), we did not detect a robust CD8^+^ T cell response induced by our scFv DNA vaccines (data not shown). One reason for that might be related to the choice of the EDIII as an antigen, as most CD8^+^ T cell epitopes are localized on the non-structural proteins, especially NS3 and NS5 (84). Our group has previously demonstrated that NS1 targeting to DCs via DEC205 induced protection partially mediated by CD8^+^ T cells (30). In addition, studies mapping CD8^+^ T cell epitopes usually use peptides of no more than 12-15 amino acids. Our peptide library consisted of 20-mers, which might have restricted the identification of CD8^+^ T cell responses.

Taken together, our results show that antigen targeting to CD8α^+^ DCs using DNA vaccines is a promising strategy to induce cellular and humoral responses and may be used in the development of more efficient dengue vaccines.

## Author Contributions

AZ and SB designed the experiments. AZ, FS, BA, HS, NF, NS, and MY conducted most of the experiments. AZ and SB analyzed the data. AZ and SB prepared the figures and wrote the manuscript. LF, DM, and DR contributed reagents. DR, LF, FS, NS, and MY revised the manuscript. All authors read and approved the final version of the manuscript.

### Conflict of Interest Statement

The authors declare that the research was conducted in the absence of any commercial or financial relationships that could be construed as a potential conflict of interest.
