# Supplementary material for: Dendritic Cell Targeting Using a DNA Vaccine Induces Specific Antibodies and CD4+ T Cells to the Dengue Virus Envelope Protein Domain III
Source: Front Immunol. 2019 Jan 29;10:59. doi: 10.3389/fimmu.2019.00059 (PMC6362411; doi:10.3389/fimmu.2019.00059)
Supplement: Supplementary file 1 [file Data_Sheet_1.PDF]

Supplementary Material

A single chain dendritic cell targeted DNA vaccine induces specific CD4^{+} T cells and antibodies to the dengue virus envelope protein domain III.

Arthur Baruel Zaneti[1]; Marcio Massao Yamamoto[1]; Fernando Bandeira Sulczewski[1]; Bianca da Silva Almeida[1]; Higo Fernando Santos Souza[1]; Natália Soares Ferreira[1]; Denicar Lina Nascimento Fabris Maeda[2]; Natiely Silva Sales[2]; Daniela Santoro Rosa[3,4]; Luís Carlos de Souza Ferreira[2]; Silvia Beatriz Boscardin[1,4*]

[1] Department of Parasitology, Institute of Biomedical Sciences, University of São Paulo, São Paulo, Brazil
[2] Department of Microbiology, Institute of Biomedical Sciences, University of São Paulo, São Paulo, Brazil
[3] Department of Microbiology, Immunology and Parasitology, Federal University of São Paulo, São Paulo, Brazil
[4] Institute for Investigation in Immunology (iii), INCT, São Paulo, Brazil

* Correspondence:
Dr. Silvia Beatriz Boscardin
sbboscardin@usp.br

Supplementary Figures

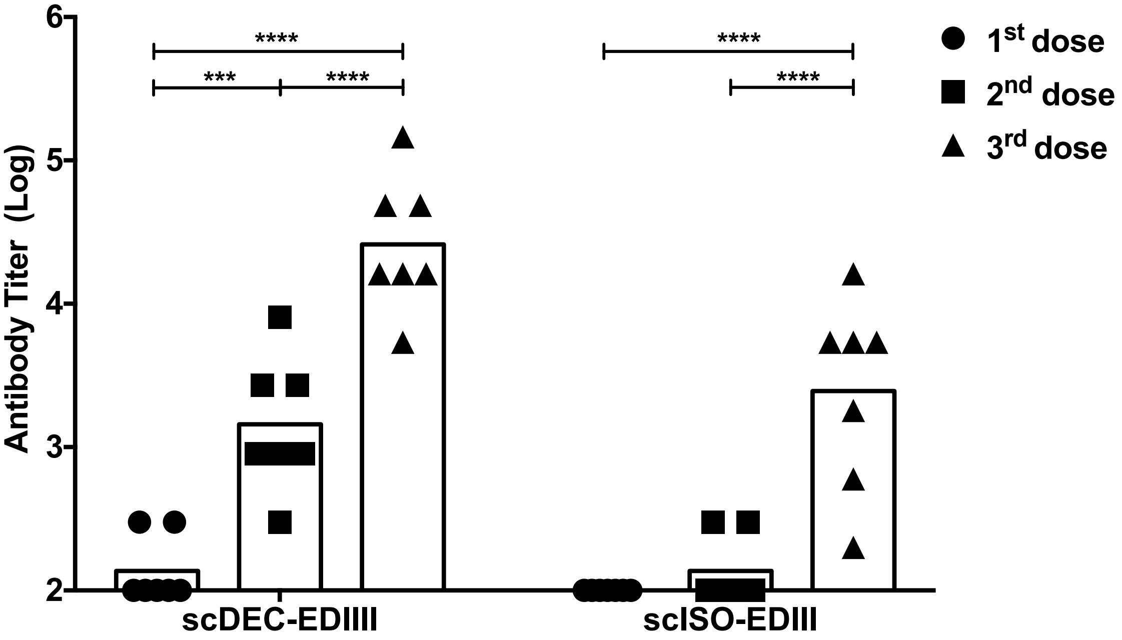

Supplementary Figure 1. Anti-EDIII antibody responses after each dose of the scDEC-EDIII or scISO-EDIII DNA vaccines. Groups of BALB/c mice (n=8) were immunized as described in Figure 2. Briefly, 100 µg of the scDEC-EDIII or scISO-EDIII plasmids were injected i.m followed immediately by electroporation. Three doses were given in 2-week intervals. Mice were bled 12 days after the 1st and 2nd dose and 14 days after the 3rd dose. ELISAs were performed using recombinant EDIII as antigen and developed using a goat anti-mouse total antibody. Antibody titers are shown in normalized log10 scale. Symbols represent individual mice, columns and bars represent the mean and SD for each group. Representative of three independent experiments. Data were analyzed by a one-way ANOVA followed by the post-test HSD Tukey. P-value indicators *** and **** refer to $p < 0.001$ and $p < 0.0001$, respectively.

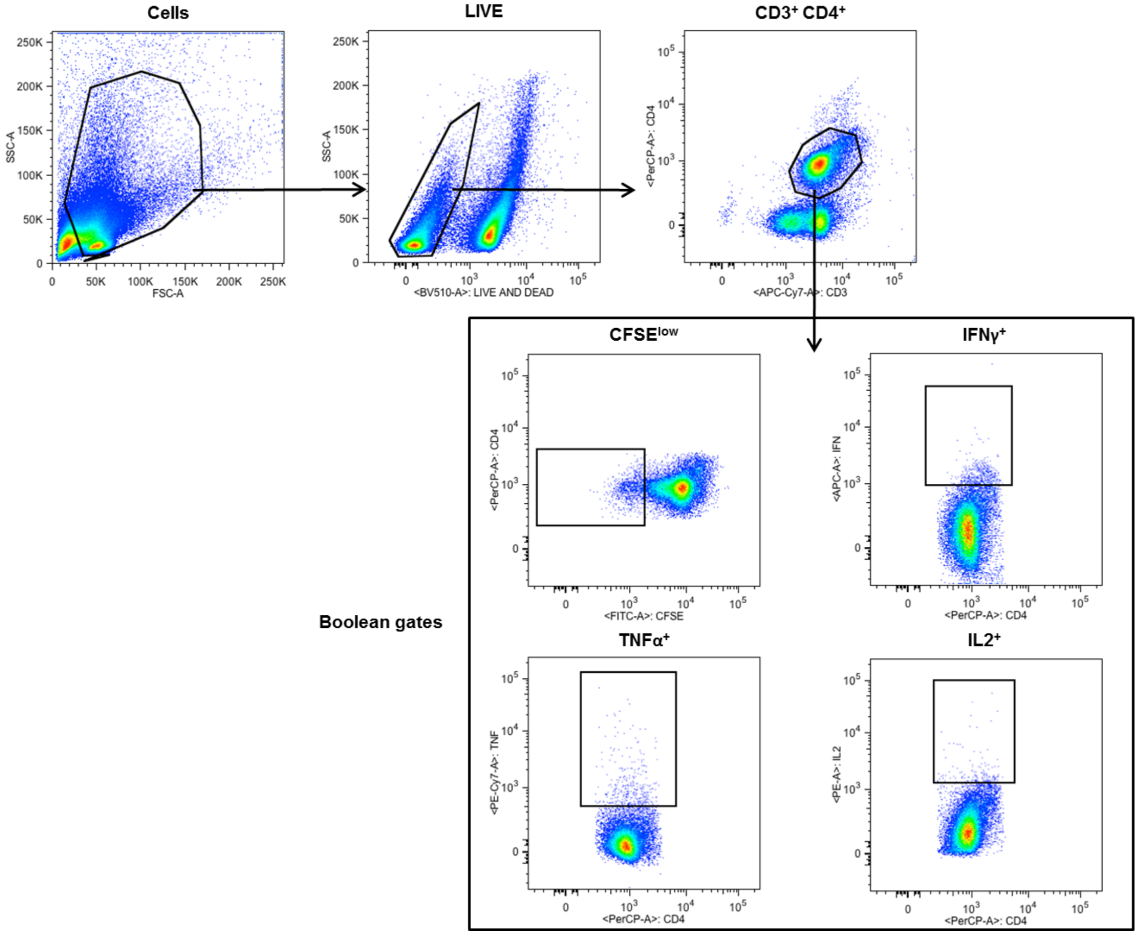

Supplementary Figure 2. Representative pseudocolor plots of the gating strategy used for the Boolean gates. Groups of BALB/c mice were immunized as described in Figure 2. Fourteen days after the administration of the last dose, mice were euthanized and total splenocytes were stained with CFSE and pulsed with 2 μg/mL of pools of peptides spanning the EDIII sequence (Table 1). After three days in culture, cells were pulsed again with the pools and αCD28 mAb and incubated for 12 h in the presence of brefeldin A. Flow cytometry was performed on the 4th day. The gating strategy is shown: size x granulosity, followed by live cells and then CD3$^+$CD4$^+$ cells. Boolean gates were applied in the CFSE^{low}, IFNγ$^+$, IL2$^+$ and TNFα$^+$ populations. Analyses were performed using FlowJo software.

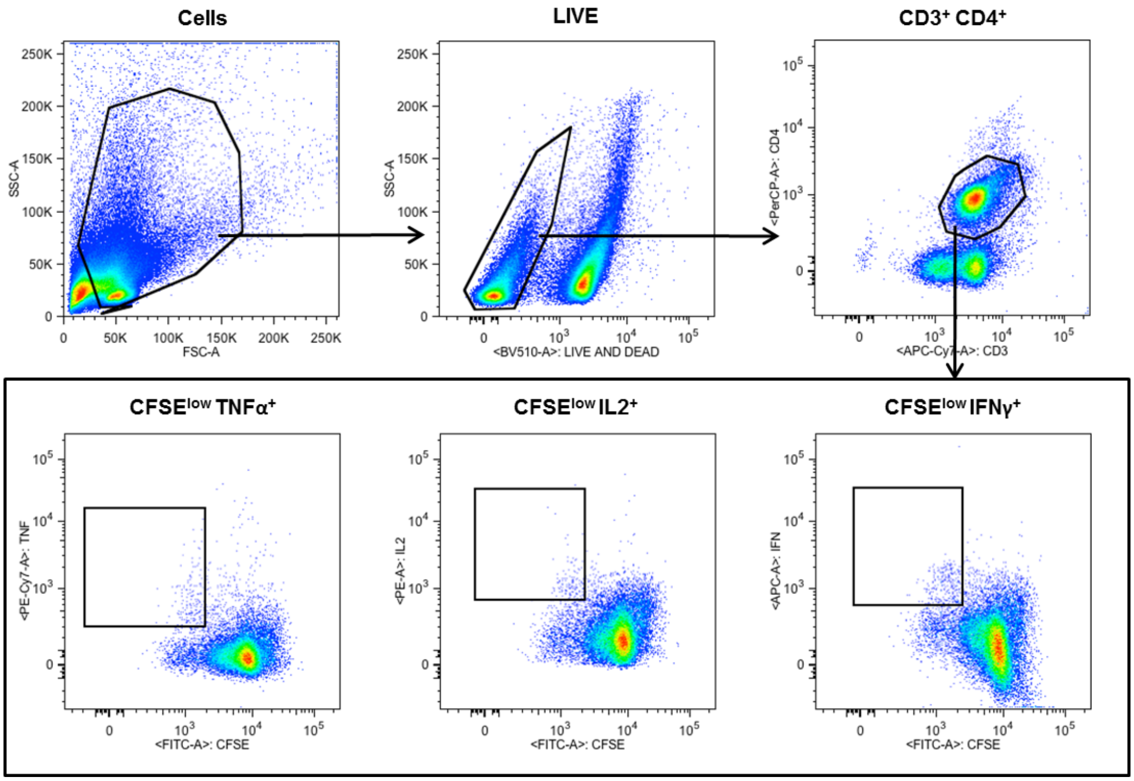

Supplementary Figure 3. Representative pseudocolor plots of the gating strategy used for intracellular cytokine production assays. Groups of BALB/c mice were immunized as described in Figure 2. Fourteen days after the administration of the last dose, mice were euthanized and total splenocytes were stained with CFSE and pulsed with 2 µg/mL of pools of peptides spanning the EDIII sequence (Table 1). After three days in culture, cells were pulsed again with the pools and αCD28 mAb and incubated for 12 h in the presence of brefeldin A. Flow cytometry was performed on the 4th day. The gating strategy is shown: size x granulosity, followed by live cells and then CD3$^+$CD4$^+$ cells. Gates in the double positive CFSE^{low}IFNγ$^+$, CFSE^{low}IL2$^+$ or CFSE^{low}TNFα$^+$ cells were used for analyses in the FlowJo software.
